# Dynamic Monitoring of CTCs and EVs to Predict Chemoimmunotherapy Efficacy in Lung Cancer

**DOI:** 10.3390/biomedicines14102161

**Published:** 2026-09-24

**Authors:** Chao Zhao, Lei Cheng, Jia Yu, Guanghui Gao, Menghang Yang, Fan Wu, Xuefei Li, Shengxiang Ren

**Affiliations:** 1 Department of Innovation and Incubation Center, Shanghai Pulmonary Hospital, Shanghai 200433, China; 2 Department of Oncology, Shanghai Pulmonary Hospital, Shanghai 200433, China; 3 Menarini Silicon Biosystem SpA, Shanghai 200433, China; 4 Department of Comprehensive of Oncology, Shanghai Pulmonary Hospital, Shanghai 200433, China

**Keywords:** circulating tumor cells, extracellular vesicles, lung cancer, immunotherapy

## Abstract

**Background:** Circulating tumor cells (CTCs), programmed cell death-ligand 1 (PD-L1) positive CTCs (PD-L1^+^CTCs) and extracellular vesicles (EVs) are important biomarkers for predicting treatment efficacy. This study comprehensively investigated the factors used to forecast chemoimmunotherapy efficacy in advanced lung cancer. **Methods:** The CellSearch system, EVAnalyzer plugin and single-cell whole-exome sequencing (WES) were used to analyze these factors. **Results:** It was found that CTCs were highly detectable in advanced lung cancer. The median number of baseline CTCs was significantly lower in non-small cell lung cancer (NSCLC) than small cell lung cancer (SCLC) (3 vs. 38, *p* < 0.0001), as were PD-L1^+^CTCs (1 vs. 5, *p* = 0.0014). PD-L1^+^CTCs decreasing after two treatment circles was associated with superior ORR. Baseline median EV counts were elevated in NSCLC compared with SCLC (11,700 vs 1371, *p* = 0.017). Patients exhibiting divergent dynamic trends between EVs and CTC/PD-L1^+^CTCs demonstrated better treatment response. The combination of CTCs, PD-L1^+^CTCs and EVs improved predictive efficacy for both NSCLC and SCLC. CTC sequencing identified multiple pathogenic somatic variants, which might affect immunotherapy efficacy. **Conclusions:** Serial dynamic monitoring of CTCs, PD-L1^+^CTCs and EVs, as well as CTC sequencing, enables the early prediction of chemoimmunotherapy response and the discovery of mechanisms that affect immunotherapy efficacy in advanced lung cancer.

## 1. Introduction

Lung cancer remains one of the most prevalent malignancies worldwide [1]. The emergence of immune checkpoint inhibitors (ICIs) has revolutionized the therapeutic landscape for both non-small cell lung cancer (NSCLC) and small cell lung cancer (SCLC). Chemoimmunotherapy has been the standard first-line treatment for NSCLC and SCLC; however, its efficacy is still limited, ranging from 43% to 74% in NSCLC [2,3] and 60–80% in SCLC [4,5]. Programmed cell death-ligand 1 (PD-L1) and tumor mutation burden (TMB) in tumor tissue exhibit limited accuracy in predicting therapeutic efficacy. Therefore, developing minimally invasive, repeatable biomarkers for real-time dynamic monitoring and early efficacy prediction is critical to individualize lung cancer chemoimmunotherapy strategies.

Circulating tumor cells (CTCs) are intact tumor cells shed from primary or metastatic lesions into peripheral blood, serving as core biomarkers in liquid biopsy research. As a critical mediator of tumor hematogenous dissemination, CTCs are closely associated with cancer progression, metastasis, and therapeutic resistance [6]. Compared with traditional tissue biopsy, CTC detection enables minimally invasive, real-time, and longitudinal monitoring of tumor dynamic changes during treatment [7,8]. CTC enumeration is not only closely associated with patient prognosis but also demonstrates significant value in therapeutic efficacy evaluation. Studies have shown that elevated baseline CTC levels indicate poor prognosis and dynamic changes in CTC counts reflect therapeutic response [9,10,11,12]. PD-L1^+^CTCs are also closely correlated with the efficacy of immune checkpoint inhibitors as well as patient survival [11,12]. However, the prediction accuracy of single-marker testing is still limited, highlighting the need for combining biomarkers to improve performance.

Several major approaches for CTC isolation, such as magnetic beads platform, microfluidic chips systems, and hybrid systems are designed and developed based on the different CTC capturing strategies. They can also be classified into positive, negative, and combined separation strategies according to the targeting cell types [13]. The first Food and Drug Administration (FDA) approved system for CTCs detection and enumeration is CellSearch^®^ (Menarini Silicon Biosystems) and widely applied in cancer research. It detects CTCs based on a combination of epithelial markers for positive selection of CTCs, and CD45 serving for immune cells elimination. We selected this system for the study.

Circulating extracellular vesicles (EVs), including exosomes, play multiple critical roles in tumor immunotherapy. EVs transport immune checkpoint ligands, oncogenic proteins and non-coding RNAs to remodel the tumor immune microenvironment, suppress anti-tumor immunity and mediate acquired resistance to ICIs [14,15,16,17,18]. Despite their biological relevance, the complementary predictive value of combined EVs and CTCs for chemoimmunotherapy response has not been systematically evaluated in lung cancer patients.

Single-cell sequencing technology has provided powerful tools for in-depth analysis of CTC heterogeneity and its relationship with immunotherapy response [19,20,21]. Combined detection of CTCs, PD-L1^+^CTCs, EVs and CTC genomic profiling may substantially enhance the accuracy of efficacy prediction. This study aims to systematically analyze the association of serial CTC, PD-L1^+^CTC and EV measurements with immunotherapy efficacy, providing new perspectives for immunotherapy of lung cancer.

## 2. Materials and Methods

### 2.1. Patients

From November 2023 to December 2024, lung cancer patients without prior systemic treatment (30 NSCLC and 23 SCLC) were enrolled. All the NSCLC patients had gene mutation detection using the ARMS method (*EGFR* Mutations Detection Kit or Five mutation gene detection kit, Amoy Diagnostics, Xiamen, China) or next generation sequencing (NGS). CTCs, PD-L1^+^CTCs, and extracellular vesicles (EVs) were detected at baseline (before cycle 1) and prior to each subsequent treatment circle. Clinicopathological features, ORR and PFS were collected in the study. The study was approved by the Ethics Committee of Shanghai Pulmonary Hospital (approval No. K20-288), and the study adheres to the STROBE guidelines.

### 2.2. CTC and PD-L1^+^CTC Detection

CTCs and PD-L1^+^CTCs were detected by CellSearch^®^ system (Menarini Silicon Biosystems, Huntington Valley, PA, USA). Briefly, 7.5 mL peripheral blood in CellSave Preservative Tubes and 6.5 mL Instrument Buffer were mixed and centrifuged in a 15 mL tube. After centrifugation, the tube was carefully placed on the CELLTRACKS^®^ Autoprep System to catch CTCs. CTCs in the cartridge were analyzed in CELLTRACKS^®^ AnalyzerII system. CTCs were defined as CD45^-^, EpCAM^+^, and cyto-keratins 8^+^, 18^+^, and/or 19^+^. PD-L1^+^CTCs assessment were performed with phycoerythrin-conjugated anti-human PD-L1 antibody, clone MIH3 (BioLegend, San Diego, CA, USA).

### 2.3. EV Quantification

The CTC images were analyzed by open-source EVAnalyzer, a Fiji plugin (version 8.2.3), to enumerate EVs [22]. It is a tool for automated, quantitative single vesicle analysis from large sets of imaging data. The parameters used in this study were as follows: Series = series1; Pixel = 0.6; Function = EV count; Thershold method= MaxEntropy; Particle size range = 0–150. The EV counts were calculated by CK^+^-DAPI^+^.

### 2.4. CTC Isolation and Single Cell Whole-Exome Sequencing

CTC isolation from cartridge was performed on DEPArray™ system. Briefly, captured CTCs were retrieved from CellSearch cartridges (Menarini Silicon Biosystems, Bologna, Italy) and transferred to 1.5 mL Protein LoBind tube by micropipette. The cartridge was washed and the suspension was transferred to the tube. The tube was centrifuged at 1000× *g* for 5 min. Supernatants were discarded, and cell pellets were resuspended and washed with DABUF buffer. The supernatant was removed and the volume was adjusted to 12 μL. The suspension was loaded onto the DEPArray™ cartridge (Menarini Silicon Biosystems, Bologna, Italy) for CTC isolation. Individual sorted CTCs were concentrated to a final volume of 1 μL and subjected to whole-exome sequencing (WES).

### 2.5. Statistical Analysis

All statistical analyses were performed using GraphPad Prism (version 8.0.1) and SPSS (version 20) software. The comparisons of CTCs, PD-L1^+^CTCs, ratios of PD-L1+ CTCs and EVs between different groups were analyzed by Mann–Whitney test. The treatment efficacies were compared by Mann–Whitney test. The Kaplan–Meier survival probability was used to analyze patients’ PFS and log-rank test was used to calculate significant differences. The relationship between CTCs or PD-L1^+^CTCs and EVs was analyzed by a nonlinear regression method. Receiver operating characteristic (ROC) curves were plotted to calculate area under the curve (AUC) values for evaluating single and combined biomarker predictive performance. The two-sided significance level was set at *p* < 0.05.

## 3. Results

### 3.1. Baseline CTC and PD-L1^+^CTC Profiles Differ Between NSCLC and SCLC

Thirty NSCLC patients and twenty-three SCLC patients were enrolled in this study. The flow chart was in Supplementary Figure S1, and the clinicopathological features of the patients were shown in Table 1. All patients were stage III-IV. Representative immunofluorescence images of CTCs and PD-L1^+^CTCs were shown in Figure 1A. CTCs were detectable in 90% (27/30) of NSCLC and 100% (23/23) in SCLC (Figure 1B). The median number of CTCs was lower in NSCLC (median = 3, range 0–120) than in SCLC (median = 38, range 1–16,722; *p* < 0.0001, Figure 1C). The median number of PD-L1^+^CTCs was also lower in NSCLC (median = 1, range 0–22) than in SCLC (median = 5, range 1–868; *p* = 0.0014, Figure 1D). The ratios of PD-L1^+^CTC in the total CTCs were not significantly different between NSCLC (median = 0.333, range 0–0.667) and SCLC (median = 0.200, range 0.0321–1; *p* = 0.081, Figure 1E). We also explored the PD-L1 expression in tumor tissues, and the consistency of PD-L1 expression in tumor tissues and CTCs (Figure 1F). Sixteen NSCLC patients had tissue PD-L1 detection in the baseline, seven of which had both CTC and PD-L1^+^CTC detection. Five were PD-L1 positive in both tissue and CTC (100%). None of the SCLC patients underwent tissue PD-L1 detection during the study period. The above results demonstrate that SCLC patients carry significantly higher circulating tumor cell and PD-L1^+^CTC burdens than NSCLC patients at diagnosis.

### 3.2. Baseline CTCs and PD-L1^+^CTCs Correlate with Chemoimmunotherapy Efficacy

We first investigated whether the CTC or PD-L1^+^CTC counts influenced immunotherapy efficacy. In the 30 NSCLC patients with chemoimmunotherapy treatment, a CTC number of less than the median value 3 achieved an ORR of 58.3% (1 disease progression (PD), 4 stable disease (SD) and 7 partial response (PR)), and of those more than 3 had an ORR of 46.7% (8SD and 7PR) (*p* = 0.441) (Figure 2A); PD-L1^+^CTCs less than the median value 1 reached an ORR of 75% (1SD, 3PR), and those more than 1 had an ORR o 66.7% (3SD, 6PR) (*p* > 0.999) (Figure 2B). Neither baseline CTC nor PD-L1^+^CTC counts demonstrated a significant association with PFS in NSCLC (Figure 2C,D). The median number of CTC and PD-L1^+^CTCs of the SCLC patients treated with chemoimmunotherapy (20 patients) were 54 and 7, separately. CTC number less than 54 had an ORR of 66.7% (1PD, 2SD and 6PR), and those more than 54 had an ORR of 55.6% (4SD and 5PR) (*p* = 0.620) (Figure 2E); PD-L1^+^CTCs less than 7 had an ORR of 44.4% (1PD, 4SD and 4PR), and those more than 7 had an ORR of 77.8% (2SD and 7PR) (*p* = 0.335) (Figure 2F). The number of CTCs as well as PD-L1^+^CTCs did not significantly influence patient PFS (Figure 2G,H). The results suggested that low a number of CTCs were associated with high ORR in both NSCLC and SCLC, and a low number of PD-L1^+^CTCs could predict high ORR in NSCLC, but not in SCLC.

### 3.3. Dynamic Decline of PD-L1^+^CTCs Predicts Chemoimmunotherapy Response

Whether the dynamic alteration of CTCs and PD-L1^+^CTCs following treatment could predict drug efficacy was investigated in the study. The patients were classified into two groups according to the number change in CTCS or PD-L1^+^CTCs: group1, declined or was stable after treatment; group2, increased after treatment. For CTC alteration in NSCLC, group2 had higher ORR than group1 (Figure 3A) either for treatment after one or two circles; for PD-L1^+^CTC alteration, group1 had the same ORR with group2 after treatment for one circle, and higher ORR than group2 after treatment for two circles (Figure 3B). For CTC alteration in SCLC, group1 had similar ORR with group2 after treatment for one circle, and higher ORR than group2 after two circles (Figure 3C); for PD-L1^+^CTC alteration, group1 had higher ORR than group2 after one and two treatment circles (Figure 3D). We also used another classification: group3, declined after treatment; group4, increased or stable after treatment (Supplementary Figure S2). These showed similar results to group1 and group2 after two circles of treatment. Collectively, two-cycle PD-L1^+^CTC decline outperformed total CTC dynamics as a predictive marker of favorable chemoimmunotherapy response across both NSCLC and SCLC cohorts.

### 3.4. EVs Could Predict Patient Response with CTCs/PD-L1^+^CTCs

EVs were analyzed based on the images (Supplementary Figure S3). Baseline EVs differed drastically between histological subgroups: median EV counts reached 11,700 in NSCLC versus 1371 in SCLC (*p* = 0.017, Figure 4A). Nonlinear regression analysis revealed a consistent inverse quantitative relationship between EVs and both total CTCs and PD-L1^+^CTCs: lower CTC burdens corresponded to elevated EV concentrations (Figure 4B,C). The ORR and PFS between EV high and low groups was not significantly different in both NSCLC and SCLC (Figure 4D,E). The alteration of EVs after one or two circles treatment had no significant difference on ORR in both NSCLC and SCLC (Figure 4F,G). We further explored whether the change in EVs with CTCs or PD-L1^+^CTCs could better predict treatment efficacy (Figure 4H). Group1 was defined as CTC or PD-L1^+^CTC had identical trend of alteration with EVs, and group2 had divergent trends. In NSCLC, group2 had higher ORR than group1 after one circle treatment for the change in CTCs/ PD-L1^+^CTCs and EVs. In SCLC, ORR was higher in group2 than group1 whether after one or two circles of treatment. The above results suggest that discordant fluctuations between EVs and CTCs/PD-L1^+^CTCs represent a stronger predictor of chemoimmunotherapy response.

### 3.5. Combination of CTCs, PD-L1^+^CTCs and EVs Achieves High Predictive AUC

Data were collected at baseline, and ROC curve analyses were drawn to predict performance of individual and combined liquid biopsy biomarkers (Figure 5 and Figure S4). Individually, CTCs, PD-L1^+^CTCs and EVs displayed weak-to-moderate discriminatory capacity for treatment response, with AUC values confined to the 0.5–0.7 range across both NSCLC and SCLC. Dual combinations (CTC + EV; PD-L1^+^CTC + EV) yielded marginal AUC improvements. Strikingly, the triple biomarker panel integrating CTCs, PD-L1^+^CTCs and EVs generated substantially elevated AUC values: 0.8095 for NSCLC (*p* = 0.1385) and 0.8052 for SCLC (*p* = 0.0333). These data confirm that multi-marker combinatorial testing markedly enhances the ability to discriminate responders from non-responders to lung cancer chemoimmunotherapy.

### 3.6. Single-Cell WES Identifies Pathogenic CTC Somatic Variants Linked to Immunotherapy

For CTCs single-cell whole-exome sequencing (WES) was conducted to find somatic variants associated with immunotherapy efficacy. Variants annotated as “likely pathogenic” and “pathogenic” were selected to analyze in the study. Four patients conducted sequencing (Table 2). MAPKAPK3, DYNC1H1, POLR1C and TRIM37 were labeled as “likely pathogenic”, and DNMT3A, CBSL, WDFY3, and EPHA7 were labeled as “pathogenic”. The dynamic alterations of CTCs, PD-L1+CTCs and EVs were presented on Figure 6. After treatment for one circle, CTCs, and PD-L1+CTCs showed the same trend with EVs in P14 and P22. After two circles, CTCs, PD-L1+CTCs and EVs showed the same trend in P16 and P23, but a different trend in P14. Clinical responses were PR for P14, P16 and P23, and stable disease (SD) for P22, highlighting the potential link between CTC genomic profiles, biomarker dynamics and therapeutic outcome.

## 4. Discussion

Chemoimmunotherapy has transformed the clinical management of advanced lung cancer; however, minimally invasive tools for early efficacy prediction remain an unmet clinical need. CTCs, PD-L1^+^CTCs and EVs are well-established liquid biopsy biomarkers for anti-tumor immunity. This study systematically characterizes their static and dynamic predictive capacity in NSCLC and SCLC patients receiving first-line chemoimmunotherapy.

Consistent with the prior literature, our cohort confirmed significantly elevated CTC and PD-L1^+^CTC burdens in SCLC relative to NSCLC at baseline, alongside high CTC positivity rates in NSCLC and SCLC [9,10,11,12,23,24,25]. Reduction in PD-L1^+^CTCs after two treatment cycles was a superior predictor of therapeutic response across both histologies, suggesting PD-L1^+^CTC subpopulations more accurately reflect the functional status of tumor cells susceptible to ICI therapy. Discrepant CTC dynamic predictive signals between NSCLC and SCLC further indicate that PD-L1^+^CTCs provides superior predictive value compared with total CTC enumeration.

EVs modulate multiple layers of tumor immune suppression and ICI resistance through encapsulated proteins, microRNAs and immune checkpoint ligands [15,26,27,28]. While prior research has validated plasma EV PD-L1 and cargo miRNAs as prognostic markers, our study demonstrates a novel inverse quantitative relationship between EV and CTCs/PD-L1^+^CTCs. Single EV quantification alone lacked predictive power for treatment response, underscoring the necessity of combining CTCs/PD-L1^+^CTCs to enhance power. Patients with divergent fluctuation trends between EVs and CTCs/PD-L1^+^CTCs exhibited higher ORR, potentially reflecting dynamic shifts in systemic tumor burden and immune signaling during therapy. This study highlights that CTCs and EVs are analyzed based on the images, which is more convenient than using different methods. Nanou et al. also analyzed CTCs and tumor-derived EVs based on the images using ACCEPT software v1.1 [29]. They found that the number of CTCs were positively associated with tdEVs. In our study, we used another software and analyzed the total EVs, which may lead to differences between the results. Moreover, this work highlights the combination of CTCs, PD-L1^+^CTCs and EVs for immunotherapy monitoring, which has not been reported before as far as we know. It provides a convenient and efficient method for predicting the efficacy of immunotherapy.

Genomic sequencing is an important method to discover more information about CTCs to understand tumor metastasis and immune tolerance or escape mechanisms [21,30,31]. The gene alterations found in the study may drive immune evasion and chemoimmunotherapy failure. For example, EPHA7 mutation patients were reported to have better efficacy and prognosis than wild type patients treated with immunotherapy [32]. It indicates that the single cell sequencing of CTCs helps to discover candidate targets for immunotherapy efficacy prediction and future mechanistic research.

Limitations of the study include but are not limited to the following: patient number, the follow up of treatment efficacy and patient prognosis, and the gene mutation function verification. Large-scale multi-center prospective cohorts are required to externally validate our findings from this study. In real clinical practice, there are some details that should be noticed. Sample acquisition time, preservation in room temperature, and detection within few hours should be strict; the analysis standards for images should be consistent; the explanation of the results should be cautious.

## 5. Conclusions

In conclusion, the study mainly found that dynamic monitoring of CTCs, PD-L1^+^CTCs and EVs can efficiently predict chemoimmunotherapy efficacy. Single-cell sequencing of CTCs is potentially beneficial for exploring the targets that affect chemoimmunotherapy efficacy and guiding clinical practice.

## Figures and Tables

**Figure 1:**
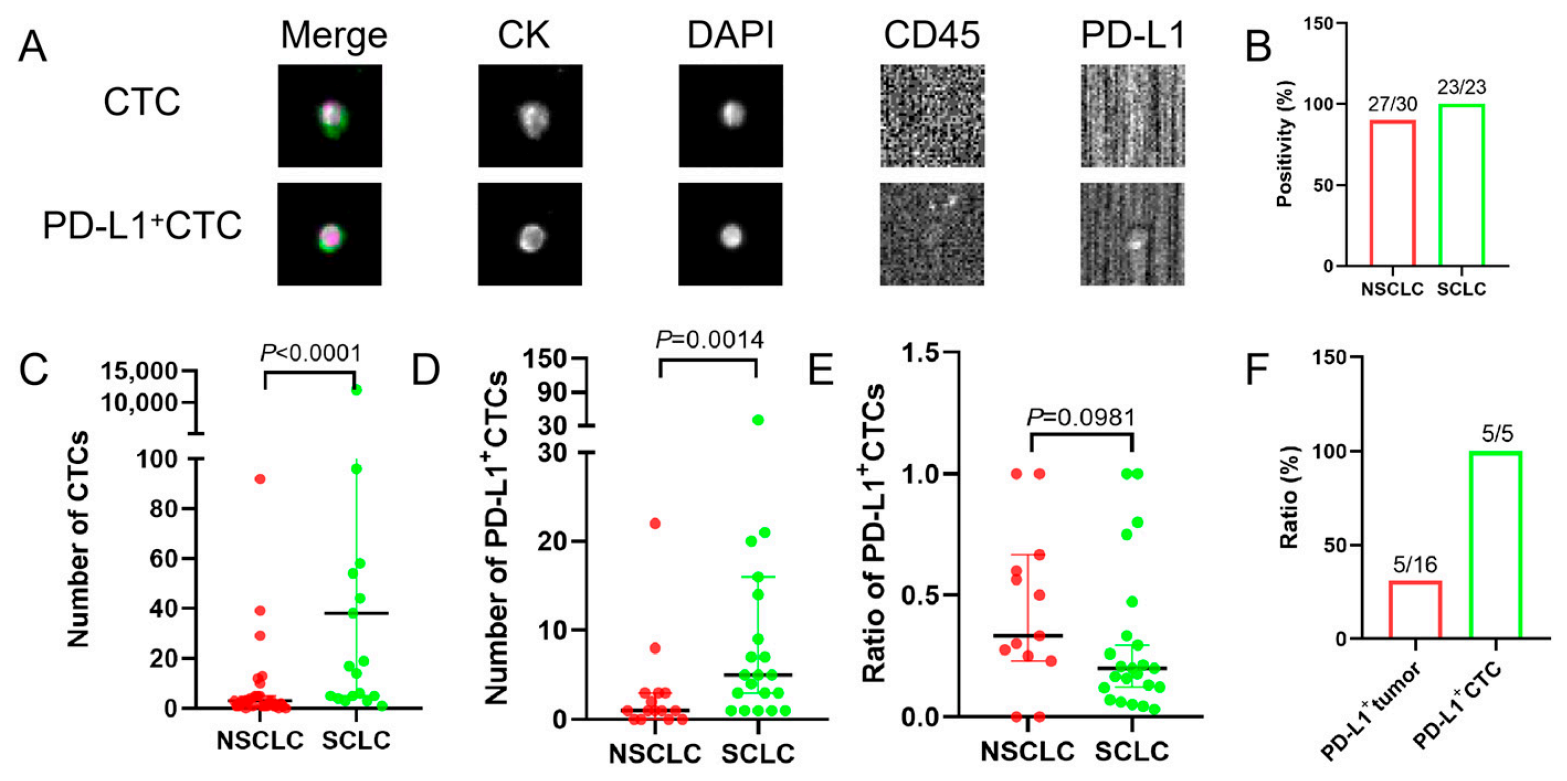
CTCs and PD-L1^+^CTCs in lung cancer. (**A**) The images of CTC and PD-L1^+^CTC in lung cancer. CTC: CK^+^/DAPI^+^/CD45^-;^ PD-L1^+^CTC: CK^+^/DAPI^+^/CD45^-^/PD-L1^+^. (**B**) The positivity of CTCs in the lung cancer patients. (**C**) The comparison of CTCs in the baseline of 30 NSCLC patients and 23 SCLC patients. (**D**) The comparison of PD-L1^+^CTCs in the baseline of 15 NSCLC patients and 23 SCLC patients. (**E**) The comparison of the ratio of PD-L1^+^CTCs in 13 NSCLC patients and 23 SCLC patients. (**F**) PD-L1 expression in tumor tissues and CTCs of baseline NSCLC. Five were both tissue and CTC positive, and two were both negative. The *p*-values were shown on the figure.

**Figure 2:**
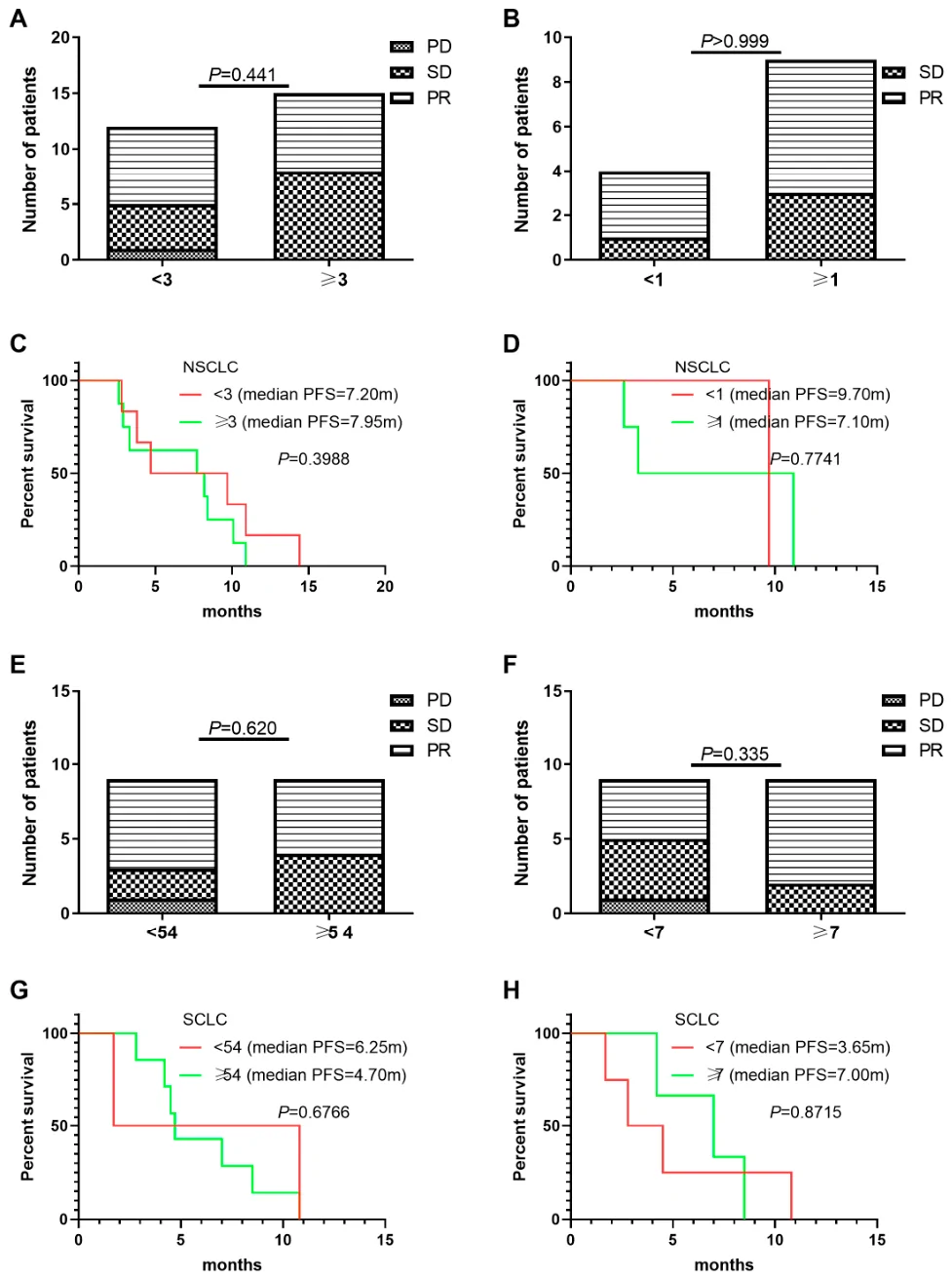
The association of CTCs as well as PD-L1^+^CTCs with treatment efficacy and PFS. (**A**) The cutoff value of CTCs in baseline NSCLC was 3. The comparison of chemoimmunotherapy efficacy was compared between patients harboring less than 3 and others. (**B**) The cutoff value of PD-L1^+^CTCs in baseline NSCLC was 1. The comparison of chemoimmunotherapy efficacy was compared between patients harboring less than 1 and others. The PFS of first-line chemoimmunotherapy was compared between the two groups (**C**,**D**). The cutoff value of CTCs in baseline SCLC treated by chemotherapy was 54, and 7 for PD-L1^+^CTCs. The treatment efficacies were compared in (**E**,**F**), and PFS in (**G**,**H**). The *p*-values were shown on the figure. PD, disease progression; SD, stable disease; PR, partial response.

**Figure 3:**
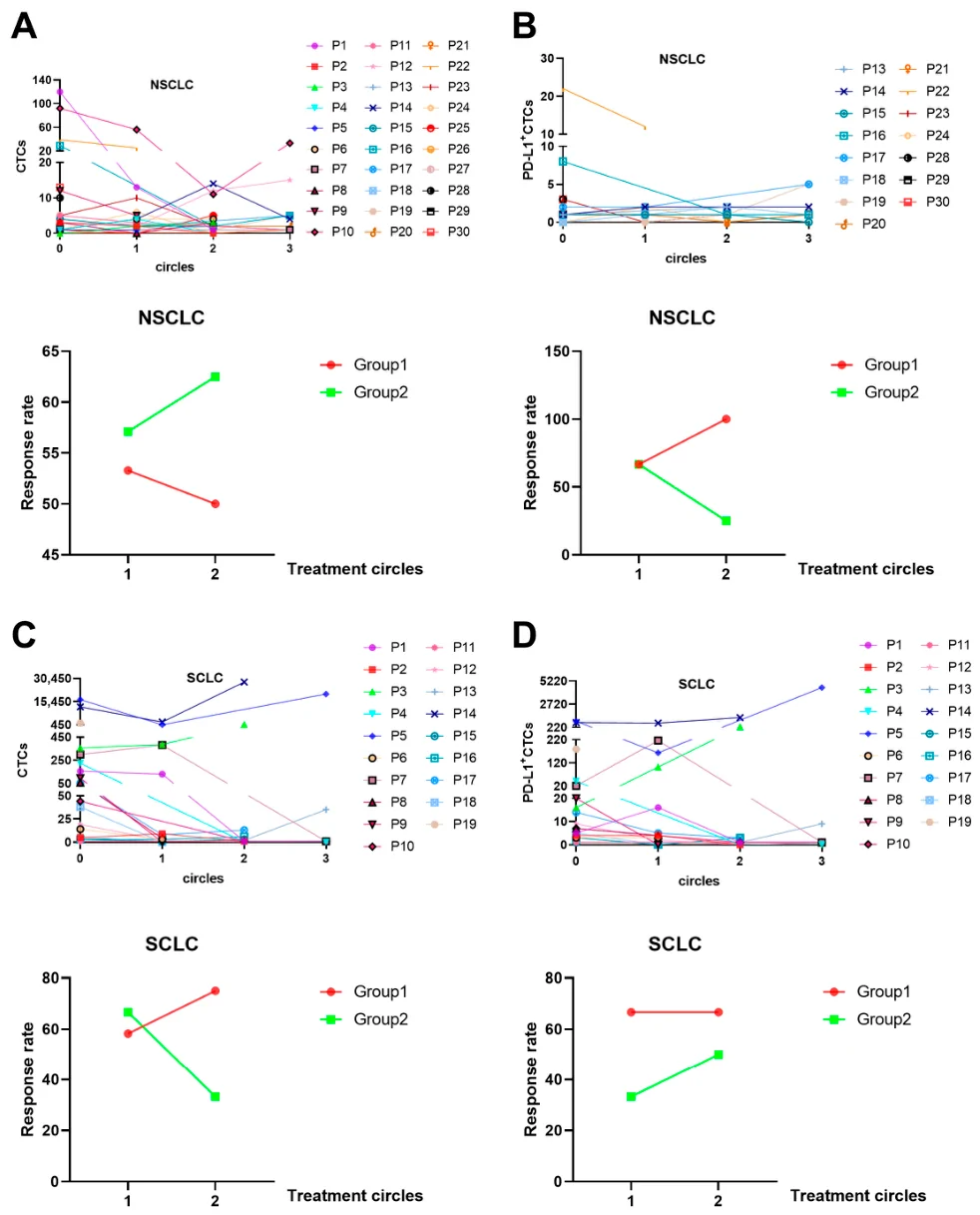
The association of dynamic alteration of CTCs and PD-L1^+^CTCs with treatment efficacy. The alteration of CTCs and PD-L1^+^CTCs in baseline (circle 0) and after treatment for different circles (circle 1/2/3) in NSCLC (**A**,**B**). Group1 was classified as the number of CTCs or PD-L1^+^CTCs decreased or equal to the baseline number, and group2 was classified as the number increased after treatment. The treatment response rates are shown below. The alteration and response rates of SCLC were shown in (**C**,**D**).

**Figure 4:**
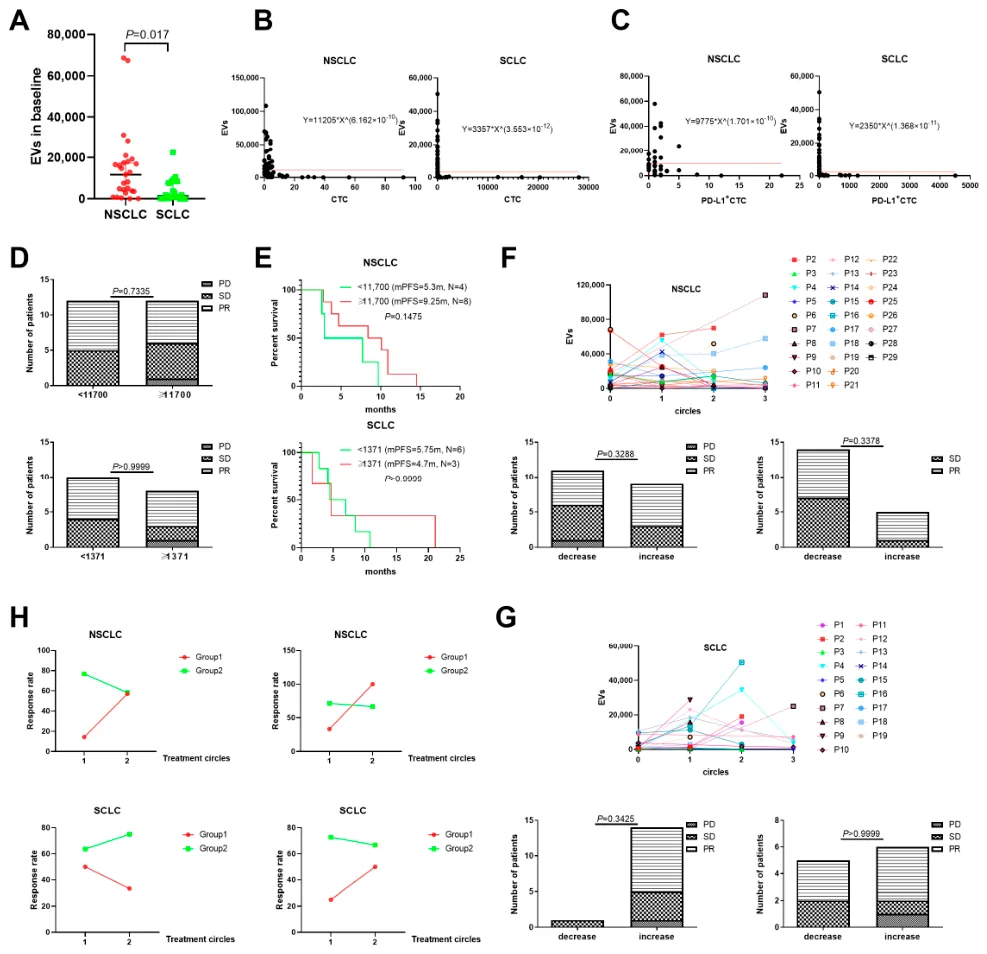
The dynamic alteration and prediction value of EVs in lung cancer. (**A**) EVs were detected in baseline of NSCLC and SCLC, and the median values were 11,700 and 1371 in NSCLC and SCLC, separately. The relationship of EVs and CTCs (**B**) as well as PD-L1^+^CTCs (**C**) was explored. (**D**) The responses in NSCLC and SCLC were compared according to the cutoff values. (**E**) The PFS was compared according to the cutoff values in NSCLC and SCLC. (**F**) The alteration of EVs during treatment in NSCLC patients. The comparison of treatment efficacy between EVs decrease and increase groups after treatment for one (**left**) and two circles (**right**). (**G**) The EVs alteration in SCLC patients, and the comparison after one (**left**) and two circles (**right**). (**H**) The response rates of the alteration of EVs and CTCs (**left**) or PD-L1^+^CTCs (**right**) were investigated in NSCLC (**up**) and SCLC (**down**). Group1 was defined as EVs had the same alteration trend with CTCs or PD-L1^+^CTCs, and group2 was defined as they had different alteration trend.

**Figure 5:**
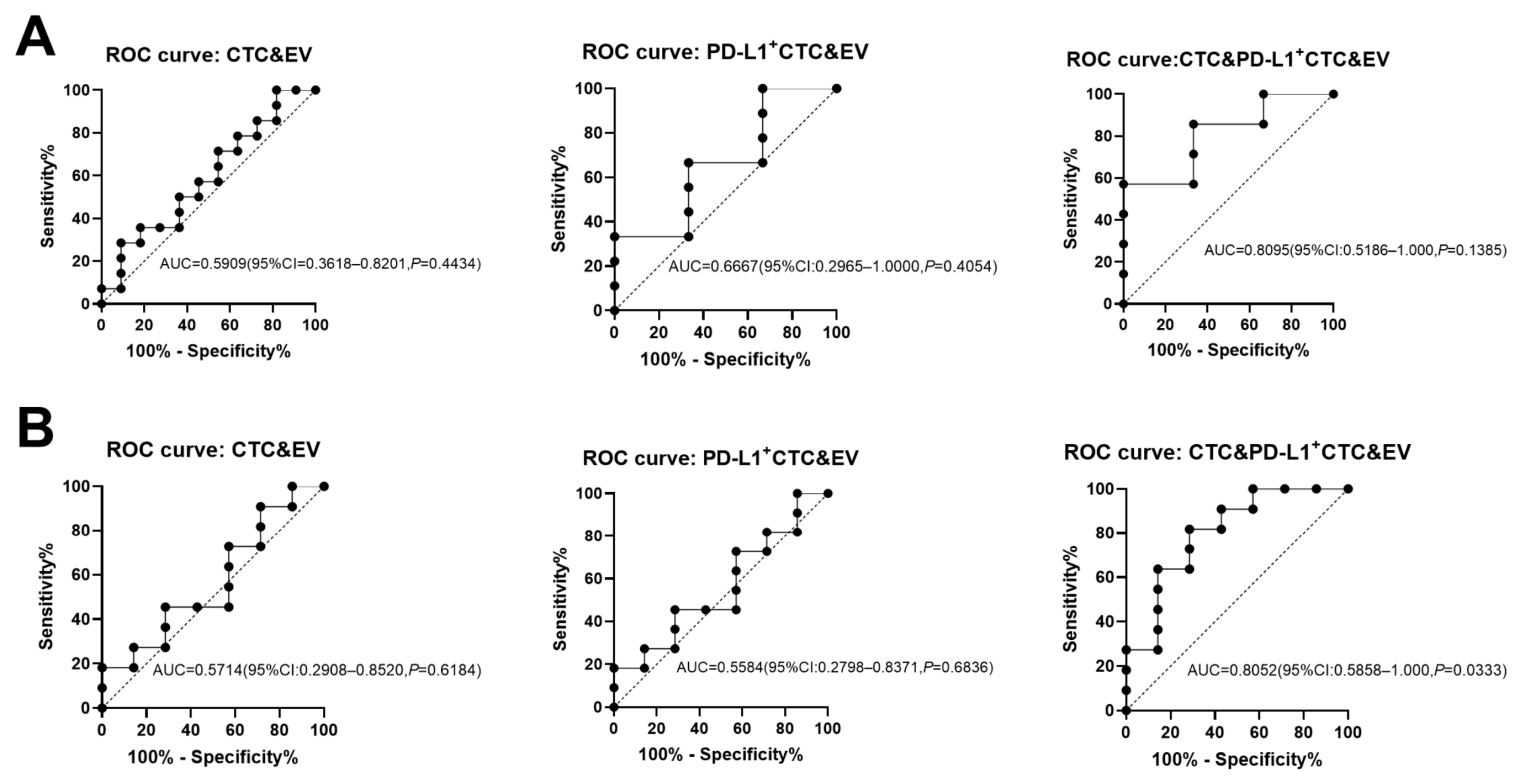
Efficacy prediction by the combination of CTCs, PD-L1^+^CTCs and EVs according to the baseline data. (**A**) The receiver operating characteristic (ROC) curves of the combination of CTC and EV, PD-L1^+^CTC and EV, and CTC, PD-L1^+^CTC and EV in NSCLC. (**B**) The ROC curves in SCLC. The combination of the three improved the AUC value.

**Figure 6:**
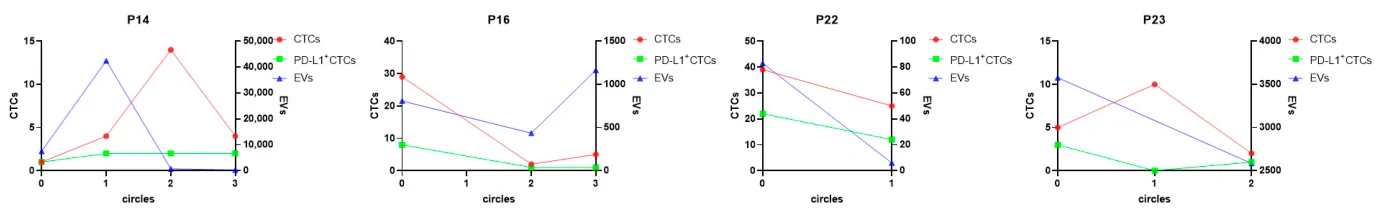
The alteration of CTCs, PD-L1^+^CTCs and EVs of the patients conducted sequencing. P14 and P16 had detection for three circles, P23 had detection for two circles, and P22 had only detection for one circle. The response of P14, P16 and P23 was PR, and P22 was SD.

**Table 1:** Clinicopathological features of the lung cancer patients in the study.

		NSCLC (n = 30)	SCLC (n = 23)
Sex	Male	27	21
	Female	3	2
Age	<65	10	8
	≥65	20	15
Smoking status	Never	19	12
	Smoking	11	11
Pathology	Adenocarcinoma	15	/
	Others *	15	/
Disease stage	I-II	0	0
	III	5	6
	IV	25	17
Gene mutation	Wild type	17	/
	*KRAS*	9	/
	Others **	4	/
Regimens	nab-paclitaxel plus carboplatin) plus PD-1 inhibitor	16	etoposide plus carboplatin plus PD-1 or PD-L1 inhibitor
	paclitaxel plus carboplatin) plus PD-1 inhibitor	2	
	pemetrexed plus carboplatin plus PD-1 inhibitor	12	
Treatment circles	>2	21	16
	≤2	9	7

*, squamous lung cancer, adenosquamous lung cancer and NSCLC not-otherwise-specified; **, including *PIK3CA*, *TP53*, *ERBB2* and *NFE2L2*.

**Table 2:** Gene mutation signature of the patients.

	P14	P16	P22	P23
Pathology	NSCLC	NSCLC	NSCLC	NSCLC
Response	PR	PR	SD	PR
Mutation gene	*DNMT3A* *CBSL*	*MAPKAPK3* *DYNC1H1*	*TRIM37*	*WDFY3* *EPHA7* *POLR1C*
CTC alteration	1-4-14-4	29-ND *-2-5	39-25	5-10-2
PD-L1+CTC alteration	1-2-2-2	8-ND-1-1	22-12	3-0-1
EV alteration	7555-42,494-742-296	808-ND-435-1165	83-6	3578-ND-2586

*, without detection.

## Data Availability

The original contributions presented in this study are included in the article/Supplementary Materials. Further inquiries can be directed to the corresponding authors.

## Supplementary Materials

The following supporting information can be downloaded at: https://www.mdpi.com/article/10.3390/biomedicines14102161/s1. Figure S1: The flow chart of the patients included in the study; Figure S2: The relationship between dynamic alterations of CTCs or PD-L1^+^CTCs and treatment efficacy; Figure S3: Typical images of EVs; Figure S4: Efficacy prediction by CTCs, PD-L1^+^CTCs and EVs.

## Abbreviations

The following abbreviations are used in this manuscript:

CTCs	Circulating tumor cells
PD-L1	programmed cell death-ligand 1
EVs	extracellular vesicles
NSCLC	non-small cell lung cancer
SCLC	small cell lung cancer
ORR	objective response rate
PD	Disease progression
SD	Stable disease
PR	Partial response
ROC	receiver operating characteristic curves
WES	whole-exome sequencing
ICIs	immune checkpoint inhibitors
TMB	tumor mutation burden
AUC	area under the curve
PFS	progression-free survival
