# Supplementary material for: Dynamic Monitoring of CTCs and EVs to Predict Chemoimmunotherapy Efficacy in Lung Cancer
Source: Biomedicines. 2026 Sep 24;14(10):2161. doi: 10.3390/biomedicines14102161 (PMC13701262; doi:10.3390/biomedicines14102161)
Supplement: Supplementary file 1 [file biomedicines-14-02161-s001.zip › biomedicines-4544423-supplementary.pdf]

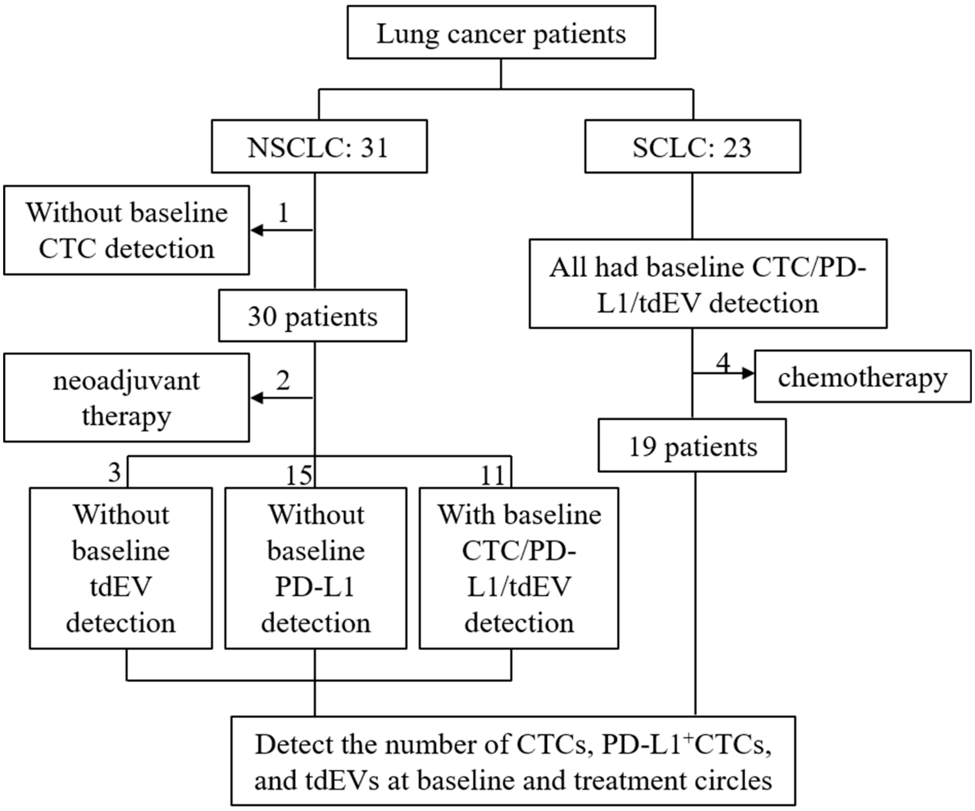

Figure S1. The flow chart of the patients included in the study.

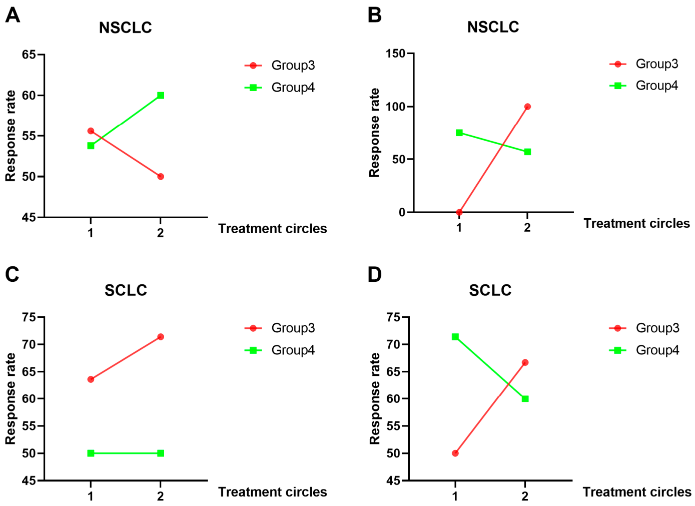

Figure S2. The relationship between dynamic alterations of CTCs or PD-L1+CTCs and treatment efficacy.

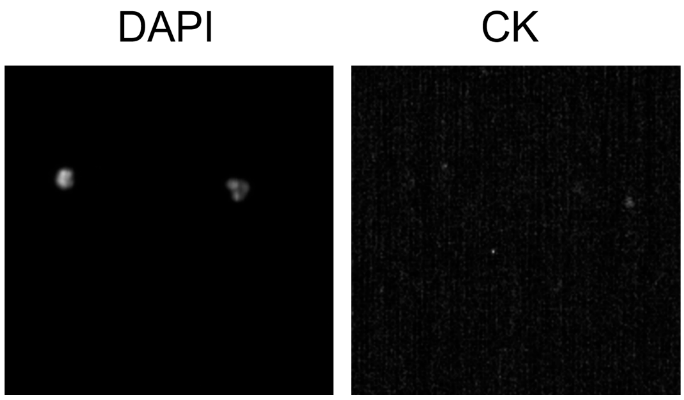

Figure S3. Typical images of EVs.

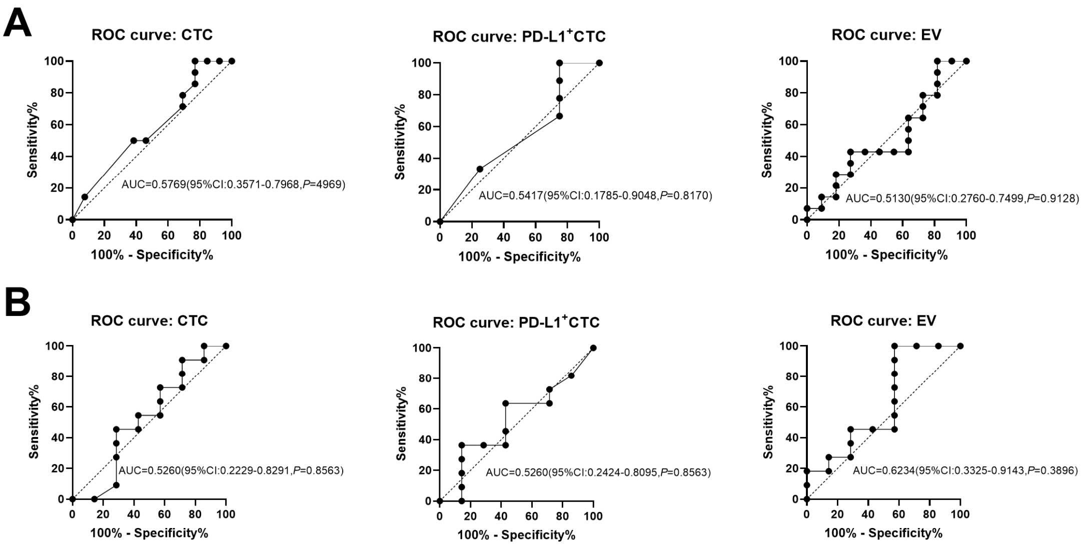

Figure S4. Efficacy prediction by CTCs, PD-L1$^+$CTCs and EVs.
